# Research on the Configuration Path of High-Quality Employment for Retired Athletes

**DOI:** 10.3390/bs16040518

**Published:** 2026-03-30

**Authors:** Chong Jiang, Dexin Zou

**Affiliations:** 1 School of Physical Education and Humanities, Nanjing Sport Institute, Nanjing 210014, China; jiang0941@163.com; 2 Academy of Sports Development and Planning, Nanjing Sport Institute, Nanjing 210014, China

**Keywords:** retired athletes, high-quality employment, capital synergy, configuration pathway

## Abstract

Achieving high-quality employment for retired athletes is essential for promoting the holistic development of athletes and accelerating the construction of a strong sports nation. From the perspective of capital collaboration, this study develops a comprehensive analysis framework by incorporating human capital, social capital, and psychological capital to systematically investigate the influencing factors and configuration pathways for high-quality employment of retired athletes. Utilizing Necessary Condition Analysis (NCA) and fuzzy-set Qualitative Comparative Analysis (fsQCA), this study discovers three main findings. First, no single condition variable independently constitutes the necessary condition for high-quality employment. Second, three configuration pathways for achieving high-quality employment are identified, including human capital–social capital synergy, human capital–psychological capital synergy, and human capital–social capital–psychological capital integration. Third, vocational skill, as a component of human capital, emerges as an important condition in configurations associated with high-quality employment. Based on the findings, this research recommends improving the athlete security policy system, promoting the accumulation of human capital, strengthening the development of psychological capital, constructing diverse social support networks, and optimizing the pathways for retired athletes to achieve high-quality employment. These aims will support retired athletes in navigating career transitions effectively while securing stable and high-quality employment.

## 1. Introduction

The term “Quality of Employment” originated from the concept of labour introduced in the international labour field during the 1990s (Sehnbruch et al., 2020). Over time, its connotation has been continuously enriched and expanded, reflecting evolving perspectives on labour standards and workplace conditions. In 1999, the International Labor Organization (ILO) proposed the concept of “Decent Work” and developed a comprehensive measurement index system (Kucera, 2007). It involves key dimensions such as employment security and standards, labour remuneration, work–life balance, job stability, social security, and skills training, providing a structured framework for evaluating employment quality (Sorensen et al., 2021). In 2001, the European Union presented the initiative to enhance the “Quality of Job” and established a systematic evaluation index system (Stefana et al., 2021). It focuses on four key dimensions, including health and social insurance benefits, skills and career development, work characteristics, and work–life harmony, which aim to provide a balanced and holistic approach to assessing job quality (Fan et al., 2021). High-quality employment refers to a state where workers, through their engagement in social labour and interaction with the means of production, experience continuous improvements in occupational safety and increasingly harmonious labour relations. Professional quality and working abilities are consistently enhanced, with education and training effectively integrated into industrial development as well as scientific and technological advancements (Spöttl & Windelband, 2021). Wage income and labour remuneration grow in tandem with economic progress, while stable employment is supported by a robust social security system (Schoukens, 2020). Furthermore, protecting rights and interests serves as a cornerstone for fostering employment stability.

Extensive scholars have conducted in-depth studies on employment quality among various groups, including migrant workers, urban youth, and retired athletes (Book et al., 2021). High-quality employment for retired athletes primarily involves access to sufficient employment opportunities, continuous improvements in labour remuneration and social security, stable job prospects, enhanced working conditions, harmonious labour relations, greater potential for career development, and higher levels of social recognition and evaluation. The career transitions of retired athletes differ significantly from those of other special groups due to the early and frequently abrupt end of competitive sport and the strong athletic identity developed over several years of training (Schmid et al., 2024). Consequently, athletic retirement is recognized as a complex lifecycle transition that contains not only disengagement from sport but also substantial changes in identity, social networks, and professional roles, all of which influence post-sport career adaptation and employment outcomes (Haslam et al., 2024). The dual-career perspective has emerged as an important framework for understanding how athletes prepare for life after sport (Robnik et al., 2022; Stambulova & Henriksen, 2025). Recent studies indicate that insufficient academic and vocational development during athletic careers can hinder employability, which creates potential challenges in post-sport career adaptation even among well-educated elite athletes (Di Rocco et al., 2025; Zhou & Zhang, 2024). In addition, cross-national evidence further highlights that engagement in dual-career pathways, which balances sport with education or employment, can facilitate smooth post-sport career transitions (Ramos et al., 2022; Thomas et al., 2024). In contrast, the absence of engagement in dual-career pathways may result in limited employment preparation and increased difficulties in post-sport career adaptation (Robnik et al., 2022; Di Rocco et al., 2025).

Existing studies on the factors influencing employment quality primarily focus on human capital, social capital, and psychological capital (Abbas et al., 2024). Schultz et al. proposed that human capital includes the entirety of knowledge, skills, and physical conditions possessed by individuals, which increases corresponding labour productivity and contributes to higher income levels as well as career development (Schultz, 1972). The accumulation of human capital plays a decisive role in shaping employment opportunities, processes, and outcomes in the labour market, making it a critical factor in achieving high-quality employment. Bourdieu et al. presented that in the absence of formal systems, social capital, defined as a collective of actual or potential resources obtained through the possession of a network of relationships, facilitates precise and comprehensive access to employment information and opportunities (Bourdieu, 2018). It enhances job stability, improves wages, and provides greater career development potential. Abundant social capital is a critical factor influencing individual employment quality, with its impact being particularly significant when human capital levels are low, thereby playing a compensatory role in promoting higher employment quality (S. Wang et al., 2023). Luthans et al. discovered that individual capital primarily consists of economic capital, human capital, social capital, and psychological capital, with psychological capital comprising elements such as confidence, hope, optimism, and resilience (Luthans & Youssef-Morgan, 2017). A positive psychological state serves as a stable resource, optimizing the capacity of individuals to adapt and overcome. It has an incremental effect on innovative work performance and job satisfaction, contributing significantly to overall employment quality. Research has demonstrated that human capital and social capital are critical factors in enhancing the employability of retired athletes (Giulianotti et al., 2021). Strong relationships, such as correlations formed with parents and relatives, play a prominent role in the job search process for retired athletes. Additionally, psychological state directly influences the corresponding career transitions and overall life satisfaction after retirement (Shander & Petrie, 2021). Human capital factors, including sports skill level, educational attainment, and vocational skills, demonstrate a significant impact on income levels (Baena-Morales & González-Víllora, 2023). Furthermore, social support networks and national employment service policies exert substantial effects on the employment prospects of retired athletes, entrepreneurial endeavours, and career development.

Existing studies on high-quality employment are primarily grounded in assumptions of independent variables and one-way linear relationships, generally relying on regression analysis and related techniques (Vaithilingam et al., 2024). The aforementioned approaches tend to neglect the synergistic effects among conditional variables and the causal complexity between the corresponding variables as well as the outcome variables. The limitation is particularly evident in research on retired athletes, whose career transitions are shaped by the simultaneous interaction of multiple resources developed during and after athletic careers. Consequently, existing studies provide limited insight into how the interaction effects of factors jointly influence the high-quality employment of retired athletes, which leaves significant gaps in understanding the multifaceted dynamics at play (Patatas et al., 2020; Voelker et al., 2022). Unlike conventional labour groups, retired athletes frequently experience heterogeneous transition pathways due to differences in educational backgrounds, vocational skills, social support networks, and psychological readiness for life after sport (Kelly & Dixon, 2014; Park et al., 2013). The interdependent resources may interact in different ways to shape diverse employment outcomes. Therefore, a configurational perspective is appropriate for examining how multiple forms of capital jointly contribute to high-quality employment among retired athletes. Recent scholars have increasingly emphasized the importance of sustainable career transitions in elite athletes. Systematic review evidence highlights that mental health support, dual-career development, and proactive identity management play critical roles in preparing athletes for post-sport careers and long-term employability (Di Rocco et al., 2025). In addition, research on dual-career development indicates that coordinated support across workplace, organizational, and policy levels is essential for balancing sport participation with education or employment opportunities (Fusco et al., 2024). Complementary studies on Career Assistance Programmes (CAPs) further demonstrate the importance of institutional support systems and resource accessibility in facilitating the career development of athletes and post-sport transitions (Hong & Minikin, 2023). The aforementioned findings emphasize the significance of examining how different forms of capital interact to influence employment outcomes among retired athletes.

**Figure 1:**
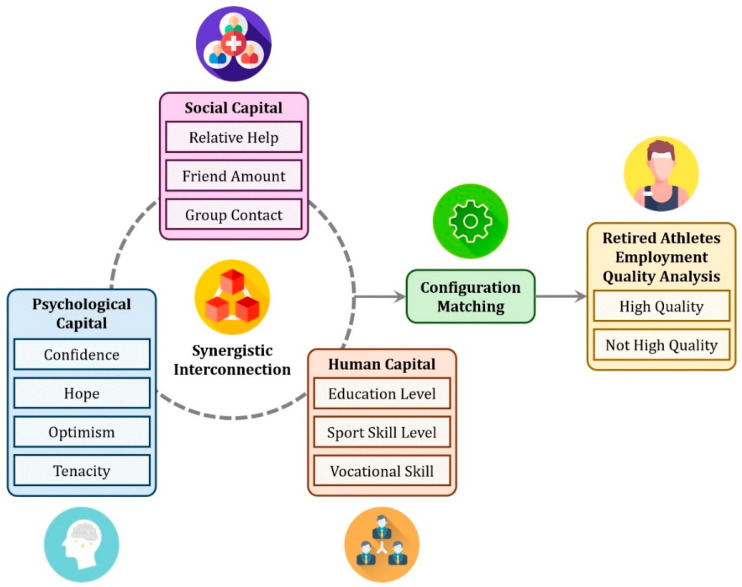
The configuration analysis framework.

From the collaborative perspective of human capital, social capital, and psychological capital, this paper constructs a configuration analysis framework for understanding the high-quality employment of retired athletes, as shown in Figure 1. Utilizing micro-survey data alongside Necessary Condition Analysis (NCA) and fuzzy-set Qualitative Comparative Analysis (fsQCA), the study investigates the influencing factors of high-quality employment for retired athletes and identifies the interaction effects among the aforementioned factors. It identifies and categorizes the pathways to achieving high-quality employment while offering decision-making references to enhance the athlete security system, providing practical insights for stakeholders.

## 2. Methods

In this section, we first outline the research technique employed in this study. Subsequently, we present a detailed description of the data source utilized for analysis. Finally, we elaborate on the processes involved in setting, measuring, and calibrating the variables central to the research.

### 2.1. Research Technique

In this paper, we adopt an innovative methodological approach that combines Necessary Condition Analysis (NCA) with the fuzzy-set Qualitative Comparative Analysis (fsQCA). NCA presents a novel perspective on causality by identifying necessary conditions and quantifying the extent to which the conditions are required for a given outcome (Dul, 2024). On the other hand, fsQCA, rooted in set theory and Boolean algebra, employs a configurational perspective to analyze how complex combinations of variables influence outcome variables (Pappas & Woodside, 2021). In recent years, fsQCA has become an extensively utilized analytical technique in disciplines such as sociology and management for addressing large-sample configuration problems through cross-case analysis (Geremew et al., 2024). The integration of NCA and fsQCA addresses the limitations of fsQCA in analyzing necessity degrees, thereby enhancing its explanatory power. Specifically, NCA complements fsQCA by providing a robust assessment of the necessity and effect size of specific conditions, while fsQCA contributes a detailed exploration of causal mechanisms and variable configurations (Ding, 2022). In this study, we first use NCA to evaluate whether specific forms of capital constitute necessary conditions for achieving high-quality employment among retired athletes and to quantify the corresponding effect sizes. Subsequently, we employ fsQCA to validate the stability of the NCA results and further investigate the causal mechanisms as well as configuration pathways through which capital accumulation influences high-quality employment (Huang et al., 2023). Prior to fsQCA, the original variables are calibrated into fuzzy-set membership scores using the direct calibration method (Ragin, 2009a, 2009b). Specifically, three qualitative anchors are specified for each condition and the outcome, including full membership, the crossover point, and full non-membership. In this study, the aforementioned thresholds are determined based on the empirical distribution of the data and relevant theoretical considerations, with the 95th percentile representing full membership, the median 50th percentile representing the crossover point, and the 5th percentile representing full non-membership. The calibration procedure allows continuous variables to be transformed into set membership scores ranging from 0 to 1, thereby capturing varying degrees of membership in the corresponding sets (Ragin, 2009a, 2009b). To ensure the robustness of the configurational research, additional robustness checks were conducted by testing alternative calibration thresholds, and the overall configurations remained largely consistent. The integrated approach provides a comprehensive analysis framework for understanding the multifaceted factors contributing to the employment outcomes of retired athletes. We provide the source code of the analysis framework in the Data Availability Statement.

### 2.2. Data Source

The data utilized in this study are derived from the “Follow-up Survey on the Employment Status of Retired Athletes in China”. The questionnaire was developed based on previously validated measurement scales related to human capital, social capital, psychological capital, and employment outcomes, and the items were adapted to the context of retired athletes in China. The instrument was further refined through expert evaluations involving scholars in sports sociology and labour economics, followed by pilot testing to ensure clarity, reliability, and contextual suitability. Each construct was measured using multiple items on a Likert-type scale, and the number of items for each construct is reported in the Data Availability Statement. The final indicators used in the analysis were computed by averaging the corresponding item scores for each construct. Reliability is assessed utilizing Cronbach’s Alpha coefficient (Taber, 2018), which yields a value of 0.872, indicating strong internal consistency. In addition, internal consistency was examined at the construct level for the multi-item measures, and all constructs satisfied the acceptable reliability threshold. Consistent with prior studies and to avoid redundancy (Greco et al., 2018; Peterson, 1994), this research reports the overall Cronbach’s alpha of the questionnaire, while the construct-level reliability results are not presented separately.

The survey targeted retired athletes across 31 provinces, municipalities, and autonomous regions in China (excluding Hong Kong, Macao, and Taiwan) who had participated in career transition training. Participants were included if they had officially retired from competitive sport and had completed or were currently participating in career transition training programmes organized by relevant sports authorities or affiliated institutions. The sampling criterion ensured that respondents had practical experience with career transition support and were able to evaluate employment-related outcomes after retirement. However, because all respondents had participated in training programmes, we acknowledge that the findings of this research should be interpreted with caution in terms of generalizability, as the sample may not fully represent all retired athletes in China who have not accessed formal transition support. The aforementioned athletes represented eight sports categories, including track and field, swimming, gymnastics, ice and snow sports, ball games, wrestling, shooting, weightlifting, and rowing. Employing stratified and quota sampling methods, a total of 1520 questionnaires were distributed. Of the questionnaires, 1471 were deemed valid, resulting in an effective response rate of 96.8%. The descriptive statistics of the sample are presented in Table 1.

### 2.3. Setting, Measuring, and Calibrating Variables

The result variable in this study is the high-quality employment level of retired athletes. Drawing inspiration from the multidimensional employment quality index framework developed by Erhel and Guergoat-Larivière (2010), we construct the employment quality index for retired athletes utilizing five sub-indicators, including income level, working hours, employment stability, social security, and career development. Income level is measured by average monthly earnings, which involves salary, performance-based commissions, bonuses, and cash benefits (Grabner & Martin, 2021). Working hours, being negatively correlated with employment quality, are represented by an inverse indicator of work intensity, derived as 1—the standardized weekly average working hours (Von Hinke Kessler Scholder, 2008). Employment stability is determined by whether a labour contract has been signed, while social security is assessed based on whether the individual is covered under the “Five Insurances and One Pension” system. Career development potential is evaluated through the job matching degree, reflecting the extent to which the transitional employment aligns with the skills of the individual and the potential for advancement (Coalter et al., 2020). The sub-indicators are standardized to ensure comparability, and the employment quality composite index Qi is calculated employing the equal-weight averaging method, following the measurement approaches of the European Commission and the European Foundation. A higher composite index value indicates a higher level of high-quality employment, as illustrated in the following equation:

$$Q_{i}=\frac{1}{5}\sum_{j=1}^{5}x_{ij}^{equ}\times 100$$

where $x_{ij}^{equ}$ refers to the index after standardization, *i* denotes individual retired athletes, and *j* represents the five sub-indicators.

This research comprehensively incorporates seven conditional variables across three dimensions, including human capital, social capital, and psychological capital. Human capital is evaluated through three variables, including education level, sport skill level, and vocational skill, reflecting the knowledge, expertise, and professional competencies of retired athletes (Erpič et al., 2004). Social capital involves bonding, bridging, and connecting dimensions, assessed utilizing the variables such as relative help, friend amount, and group contact, which capture the strength of close relationships, broader social networks, and ties to institutional resources, respectively (Linnér et al., 2022). Psychological capital is measured employing the psychological capital questionnaire developed by Ke and Ding (2018), which extensively evaluates confidence, hope, optimism, and resilience as secondary indicators, with the corresponding arithmetic average serving as the overall measure of psychological capital (Kim et al., 2020). Other conditional variables are quantified on a five-point Likert scale (Mercier et al., 2023). Information on measurement approaches is presented in Table 2.

The reliability of the questionnaire was assessed using Cronbach’s alpha coefficient. The experimental results indicate that Cronbach’s alpha values for all latent variables exceeded 0.70, which suggests acceptable internal consistency of the scale and satisfactory reliability, as illustrated in Table 3.

Calibration involves assigning membership scores for each case within the set. For this research, the continuous data samples are calibrated as fuzzy sets following the standard direct method proposed by Ragin (2014). Based on prior studies, the calibration thresholds for both the outcome variable and the condition variables of high-quality employment are established as follows: the 95% of the sample data represents full membership, the median serves as the crossover point, and the 5% indicates no membership (Pappas & Woodside, 2021). Non-high-quality employment is calibrated as the complement of the high-quality employment set. The descriptive statistics and calibration details for the outcome and condition variables are summarized in Table 4.

## 3. Results

In this section, we first present a discussion of the experimental results from the necessary condition analysis, highlighting key factors that influence high-quality employment. Subsequently, we conduct an adequacy analysis to identify sufficient conditions for achieving high-quality employment among retired athletes. Finally, we perform a robustness test to validate the stability and reliability of the experimental results under varying analytical thresholds.

### 3.1. Necessary Condition Analysis

A necessary condition refers to a factor that must be present for a particular outcome to occur (Liao et al., 2010). In this study, calibrated data are analyzed to determine the effect size and bottleneck level of necessary conditions affecting high-quality employment, thereby identifying which factors may qualify as necessary conditions. Two estimation methods, Ceiling Regression (CR) and Ceiling Envelopment (CE), are employed to calculate effect sizes (H. Wang et al., 2022). According to the NCA method, necessary conditions are indicated by a significant effect size (Dul, 2016), where d ≥ 0.1, and significant results from Monte Carlo simulation replacement tests, where *p* ≤ 0.01.

The analysis results indicated that educational level (*p* = 0.5), sports skill level (*p* = 1.0), relative help (*p* = 1.0), and group contact (*p* = 0.3) are not statistically significant. Although vocational skill (*p* = 0.010), friend amount (*p* = 0.001), and psychological capital (*p* = 0.009) demonstrate statistically significant effects, the corresponding effect sizes are relatively small and thus do not meet the threshold to be considered strictly necessary conditions for high-quality employment (McNamara et al., 2022), as illustrated in Table 5. Our findings suggest that while certain factors may have measurable influences, no single condition meets the criteria to be deemed essential for achieving high-quality employment.

The bottleneck level represents the minimum threshold necessary for a specific outcome to occur (Peery et al., 2012). In this study, all variables are considered continuous, and the CR estimation method is developed to analyze the bottleneck levels. The experimental results indicate that achieving high-quality employment at the 80% level requires only psychological capital at the necessary threshold. However, when the quality of employment reaches 100%, multiple conditions jointly contribute, including 25.6% education level, 51.4% vocational skill, 47.8% friend amount, 13.7% group contact, and 24.7% psychological capital, as detailed in Table 6. The experimental results underscore that high-quality employment is not determined by any single factor. Instead, it emerges from the organic combination of multiple conditions. Furthermore, as the quality of employment improves, both the level and the complexity of the necessary conditions increase, which emphasizes the multifaceted nature of achieving high-quality employment outcomes.

To further validate the necessary conditions, we employ the fsQCA method to examine the corresponding consistency of individual conditions. The experimental results illustrate that the consistency of any single condition is below the threshold value of 0.90, as displayed in Table 7. The aforementioned findings align with the analysis results of the NCA method. Collectively, the experimental results confirm that no single condition qualifies as necessary for achieving high-quality employment, which reinforces the requirement to investigate the configurational interplay among multiple forms of capital.

### 3.2. Adequacy Analysis of High-Quality Employment for Retired Athletes

**Figure 2:**
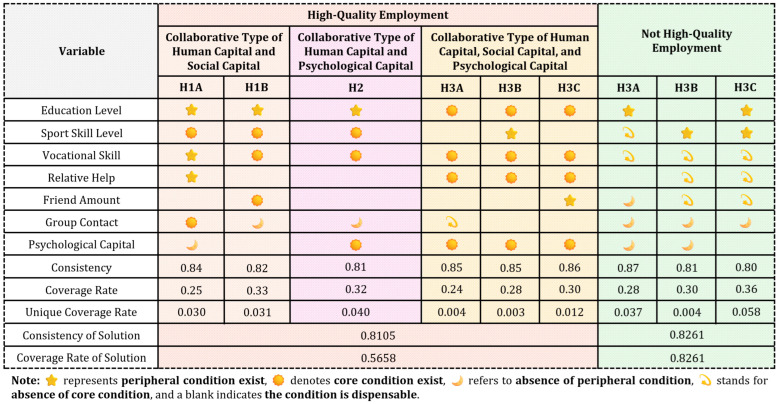
The configurations sufficient for high-quality employment and non-high-quality employment. Specifically, **peripheral conditions exist** indicates the condition supports the configuration but is less central to the causal pathway. **Core conditions exist** indicates the condition plays a central role in the configuration leading to the outcome and appears consistently across simplified solution terms. **Absence of a peripheral condition** indicates the condition is absent but plays a secondary or supporting role in the configuration. **Absence of a core condition** indicates that the lack of this condition forms a central component of the configuration associated with the outcome. A blank cell signifies that the condition is not relevant or not required in the corresponding configuration, which indicates the outcome can occur regardless of whether this condition is present or absent.

When a condition or configuration is present and consistently leads to a specific outcome, it is considered a sufficient condition for the corresponding outcome. In this study, the criteria proposed by Ragin are applied (Ragin, 2014), setting the original consistency threshold at 0.80, the PRI consistency threshold at 0.70, and the sample frequency threshold at 10. Leveraging the fsQCA method, this study derived complex solutions, intermediate solutions, and reduced solutions, selecting the intermediate solutions to interpret the configuration pathways for achieving high-quality employment. Conditional variables identified in both the intermediate and reduced solutions are regarded as core conditions, signifying their critical impact on high-quality employment (Liao et al., 2010). In contrast, conditional variables that appear only in the intermediate solution are treated as edge conditions, indicating the corresponding peripheral conditions for high-quality employment (Liao et al., 2010). The aforementioned results are detailed in Figure 2, highlighting the core and edge conditions that collectively shape the configuration pathways to high-quality employment.

From the analysis of individual configurations, the consistency levels of all six configurations that lead to high employment quality exceed the threshold of 0.80, confirming that each configuration is a sufficient condition for high employment quality (Fu et al., 2024). On a broader scale, the overall solution consistency is 0.81, which is also above the 0.80 threshold, indicating that the combined configurations collectively qualify as sufficient conditions for achieving high employment quality. The overall coverage is 0.65, demonstrating that the corresponding six configurations provide strong explanatory power for the mechanisms underlying high employment quality. Additionally, the analysis identified three capital configurations associated with non-high-quality employment, further emphasizing the diverse pathways and factors influencing employment outcomes.

### 3.3. Robustness Test

To ensure the robustness of the results, we apply a robustness testing method specific to the set theory employed in fsQCA. By raising the consistency threshold from 0.80 to 0.85, and the sample frequency threshold at 15, we conduct the configuration analysis again under stricter conditions (Pappas & Woodside, 2021). It results in four sets of solutions, with the overall solution consistency increasing to 0.8396, while the coverage decreases to 0.5299, aligning with theoretical expectations. The subset relationship between the results at the two consistency thresholds confirms the robustness of the research conclusions, demonstrating that the findings remain stable under varying analytical criteria.

## 4. Discussion

We discuss the capital configurations associated with both high-quality employment and non-high-quality employment among retired athletes.

### 4.1. Capital Configuration Leading to High-Quality Employment of Retired Athletes

Based on the core conditions, the six configurations leading to high-quality employment of retired athletes can be categorized into the following three categories.

The first category is the human capital–social capital synergy pathway. Configurations H1A and H1B demonstrate that high-quality employment can be achieved when sport skill level or vocational skill, together with friend amount or group contact, function as core conditions, complemented by cultural education or relative help as peripheral conditions. In the aforementioned pathway, human capital constitutes the fundamental element for enhancing the overall quality of employment. The sport skill and vocational skill of athletes mutually compensate for the human capital deficits resulting from career transitions after retirement. Social capital serves as an important complementary mechanism for resource allocation, which provides individuals with channels to obtain employment opportunities and improve employment quality (Ray et al., 2023). Social capital has been referred to as “the capital of the poor” due to its significant role in supporting the employment prospects of vulnerable groups (Zhang & Zhao, 2024). In the context of labour market asymmetries and relational social structures, retired athletes may leverage the corresponding network connections, such as the bridging role of friendships and the linking role of associations as well as organizations, to acquire diverse employment information and external resources. This compensates for resource deficiencies and enhances the competitiveness of retired athletes in the job market.

The second category is the human capital–psychological capital synergy pathway. Configuration H2 indicates that high-quality employment can be achieved when sport skill, vocational skill, and psychological capital function as core conditions, with cultural education serving as a peripheral condition. With the widespread availability of higher education, cultural education alone may no longer constitute a distinct competitive advantage (Lumby & Foskett, 2016). Instead, sport skill and vocational skill emerge as critical factors in securing employment opportunities and enhancing employment quality (De Schepper & Sotiriadou, 2018). Additionally, the retirement transition represents a significant transition for the careers of athletes. High levels of self-efficacy, optimism, hope, and resilience may help alleviate the sense of “relative deprivation” that can accompany the aforementioned transition (Hassan et al., 2024; Bamberg et al., 2018). The psychological capital enables retired athletes to approach career uncertainties and related pressures with a proactive as well as adaptive mindset. By leveraging the collaborative effects of human capital and psychological capital, retired athletes may better realize the value of sport skill and vocational skill. This synergy not only facilitates their personal adjustment and resilience as athletes but also strengthens their capacity to adapt to post-retirement careers, thereby contributing to high-quality employment outcomes.

The third category is the human capital, social capital, and psychological capital synergy pathway. Configurations H3A, H3B, and H3C demonstrate that high-quality employment can be achieved when education level, vocational skill, relative help, and psychological capital function as core conditions, while sport skill and friend number serve as peripheral conditions. Prior research highlights the synergistic relationship among human capital, social capital, and psychological capital, which suggests that the corresponding factors mutually influence and reinforce each other (Song et al., 2024; Miao et al., 2022). Collective development may contribute to improved work performance and employment quality. For retired athletes, vocational skill and cultural education represent foundational elements for attaining stable and high-quality employment opportunities. In relational social contexts, the effective utilization of “strong relationship” social capital, such as that derived from kinship and familial ties, may facilitate access to employment resources and support career development (Tsai, 2021). Moreover, positive psychological capital may empower individuals to maximize their potential and make effective use of available social support. Therefore, for retired athletes, the integration of the aforementioned three forms of capital supports career transitions, enabling retired athletes to overcome challenges and achieve high-quality employment outcomes.

Additionally, a horizontal analysis of the core conditions across the six configurations reveals that vocational skill is present in all six configurations, whereas psychological capital appears in four configurations. The finding suggests that advanced vocational skill, as a form of human capital, and psychological capital frequently co-occur across multiple configurations associated with high-quality employment among retired athletes. The recurrent presence across different configurations indicates the potential significance in facilitating successful career transitions and contributing to improved employment outcomes.

### 4.2. Capital Configuration Leading to Non-High-Quality Employment of Retired Athletes

The analysis identified three configurations associated with non-high-quality employment, as illustrated in Figure 2. First, configuration NH1 indicates that the absence of sport skill and vocational skill as core conditions constitutes the primary mechanism leading to non-high-quality employment. In addition, the absence of several peripheral conditions, including friend amount, group contact, and psychological capital, further contributes to the outcome of non-high-quality employment. Even when cultural education appears to be a peripheral condition, it does not compensate for the lack of these core conditions. Second, configuration NH2 suggests that, in the absence of vocational skill, social capital, and psychological capital, high-quality employment remains difficult to achieve. Although sport skill is present as a peripheral condition, its presence alone does not offset the absence of vocational skill and other complementary resources. Third, configuration NH3 highlights that the absence of vocational skill and social capital as core conditions represents the central pathway associated with non-high-quality employment. In this configuration, cultural education and sport skill function as peripheral conditions. A horizontal analysis across the three configurations associated with non-high-quality employment reveals that the absence of vocational skill consistently appears as a core condition in all three configurations, which suggests that insufficient vocational skill frequently characterizes pathways associated with non-high-quality employment. Overall, the aforementioned finding indicates that limitations in vocational skill may substantially constrain employment outcomes, particularly when accompanied by deficiencies in other forms of capital.

### 4.3. Implications and Limitations

Beyond the general perspectives of high-quality employment and capital theory, the findings of this study contribute to the growing literature on athlete retirement and career transition. Previous studies have highlighted that retirement from elite sport frequently involves complex adjustments in identity, social networks, and career orientation (Di Rocco et al., 2025; Schmid et al., 2024). The present findings provide additional evidence that successful employment outcomes after athlete retirement are shaped by the joint interaction of multiple forms of capital, rather than by any single condition in isolation. This finding is consistent with studies emphasizing the multidimensional nature of athlete career transitions, which involve educational preparation, psychological adaptation, and access to supportive networks (Stambulova & Henriksen, 2025; Park et al., 2013). In particular, the recurrent presence of vocational skill and psychological capital across several configurations supports prior research which suggests that career preparedness and psychological adaptability are important factors in facilitating post-sport career development. Earlier studies have shown that athletes who cultivate transferable vocational skills and maintain strong psychological resilience tend to adapt effectively to the labour market demands after retirement (Yiapanas, 2024; Zhou & Zhang, 2024). Our findings extend recent studies by demonstrating that the aforementioned resources operate in combination with other forms of capital, thereby generating multiple pathways toward employment outcomes. Furthermore, our findings complement existing research on dual-career development and transition support systems. Previous studies have emphasized that structured support programmes, educational opportunities, and institutional guidance can play important roles in preparing athletes for life after sport (Brassard et al., 2024). The findings of this research suggest that the aforementioned support mechanisms may be particularly beneficial when they facilitate the simultaneous accumulation of diverse forms of capital, including vocational competence, social resources, and psychological capacity.

Our findings should be interpreted in light of several limitations. First, this research relies on cross-sectional data capturing employment outcomes of retired athletes at a single point in time. Accordingly, the analysis cannot fully reflect the dynamic process of career transition following athletic retirement. Second, the variables in this research are primarily measured utilizing self-reported survey responses. Although reliability and validity assessments indicate acceptable measurement quality, self-reported responses may still be influenced by subjective perceptions or response bias. Third, the empirical context of this research is institutionally and culturally specific to China. The Chinese elite sport system, including its training structures and transition support policies, differs from those in many other countries. Therefore, caution is warranted when generalizing the findings of this research to other institutional contexts. Fourth, the sample consists of retired athletes who have participated in career transition training programmes. While this sampling approach ensures that respondents have experience with structured transition support, it may limit the broader representativeness of the sample. Retired athletes who have not accessed such programmes may encounter different transition experiences and challenges. Finally, although configuration analysis enables the identification of complex relationships among different conditions, this research does not permit strong causal inference. The identified configurations should therefore be interpreted as patterns associated with employment outcomes rather than definitive causal mechanisms. Future works could address the aforementioned five limitations by adopting longitudinal designs, incorporating multi-source data, and examining cross-national samples to comprehend how institutional environments shape post-sport career development of athletes.

## 5. Conclusions

This study examines the impact of the interaction among human capital, social capital, and psychological capital on the employment quality of retired athletes through a configuration perspective utilizing the fsQCA method, with nearly 1500 retired athletes as the research sample. The three main conclusions are as follows. First, human capital, social capital, and psychological capital alone cannot constitute the necessary conditions for either high-quality or non-high-quality employment. However, vocational skill was present across all configurations associated with high-quality employment, suggesting that it may be an important enabling condition within favourable capital combinations to support retired athletes in achieving high-quality employment. Second, the factors influencing employment quality are characterized by multiple configurational pathways, leading to three distinct pathway categories for achieving high-quality employment, including the human capital–social capital synergy type, the human capital–psychological capital synergy type, and the human capital–social capital–psychological capital integration type. Finally, vocational skill is present in all configurations associated with high-quality employment, while the absence of vocational skill consistently appears in configurations linked to non-high-quality employment. This study highlights the potentially important role of vocational skill training within favourable capital combinations in enhancing the employment quality of retired athletes and facilitating their successful career transitions.

## Figures and Tables

**Table 1:** Variable descriptive statistics.

Question Item	Subquestion Item	Frequency (N)	Proportion (%)	Question Item	Subquestion Item	Frequency (N)	Proportion (%)
Gender	Male	730	49.6	Employment Area	Eastern Part	402	27.3
	Female	741	50.4		Middle Part	327	22.2
Age	Over 35 Years Old	93	6.3		Northeast Part	529	36.0
	From 31 to 35 Years Old	259	17.6		East Part	213	14.5
	From 26 to 30 Years Old	575	39.1	Sports Event	Track and Field	221	15.0
	Under 26 Years Old	544	37.0		Swimming	53	3.6
Sport Skill Level	International Elite Athlete	116	7.9		Gymnastics	36	2.5
	Elite Athlete	517	35.1		Ice and Snow Sports	69	4.7
	First Grade Athlete	521	35.4		Ball Games	336	22.8
	Second Grade Athlete	317	21.6		Wrestling	123	8.4
Education Level	Master’s Degree or Above	109	7.4		Shooting	59	4.0
	Undergraduate and Junior College	1215	82.6		Weightlifting, Rowing, etc.	574	39.0
	High School, Technical Secondary School, or Below	147	10.0				

**Table 2:** The measurement of results and conditional variables.

Types of Variables		Variable Name	Measurement
Outcome Variable		Quality of Employment	Comprehensive index of high-quality employment for retired athletes. $Q_{i}$
Conditional Variable	Human Capital	Education Level	Master Degree = 5; Undergraduate and Junior College = 4; High School and Technical Secondary School = 3; Junior High School = 2; Elementary School and Below = 1.
		Sport Skill Level	International Elite Athlete = 5; Elite Athlete = 4; First Grade Athlete = 3; Second Grade Athlete = 2; Others = 1.
		Vocational Skill	Very Strong = 5; Relatively Strong = 4; Average = 3; Worse = 2; Very Bad = 1.
	Social Capital	Relative Help	Great = 5; Relatively Great = 4; Average = 3; Relatively Few = 2; Few = 1.
		Friend Amount	Great = 5; Relatively Great = 4; Average = 3; Relatively Few = 2; Few = 1.
		Group Contact	Great = 5; Relatively Great = 4; Average = 3; Relatively Few = 2; Few = 1.
	Psychological Capital	Psychological Capital	Have the Confidence to Pursue New Life Goals (25%); Willing to Take on Challenging Work (25%); Always Look on the Bright Side of Things (25%); Able to Recover Quickly from Setbacks (25%). Assign Value Separately: Fit Well = 5; Fit = 4; Average = 3; Relatively Not Fit = 2; Not Fit At All = 1.

**Table 3:** The reliability analysis of the questionnaire.

Types of Variables	Variable Name	Factor Loading	Cronbach’s Alpha Coefficient
Quality of Employment	Income Level	0.763	0.782
	Working Hours	0.778	
	Employment Stability	0.810	
	Social Security	0.809	
	Career Development	0.769	
Human Capital	Education Level	0.794	0.811
	Sport Skill Level	0.831	
	Vocational Skill	0.773	
Social Capital	Relative Help	0.819	0.791
	Friend Amount	0.728	
	Group Contact	0.797	
Psychological Capital	Have the Confidence to Pursue New Life Goals	0.832	0.827
	Willing to Take on Challenging Work	0.846	
	Always Look on the Bright Side of Things	0.799	
	Able to Recover Quickly from Setbacks	0.783	

**Table 4:** The descriptive statistics and calibration of the outcome and condition variables.

	Descriptive Statistics				Fuzzy-Set Calibration		
Variable	Mean Value	Standard Deviation	Maximum Value	Minimum Value	Full Membership	Intersection	No Membership
Employment Quality Index	51.94	0.581	98.0	4.0	83.92	53.2	12.57
Education Level	3.95	0.481	5	2	5	4	3
Sport Skill Level	3.22	1.027	5	1	5	3	1
Vocational Skill	3.89	1.193	5	1	5	4	2
Relative Help	2.37	1.28	5	1	5	3	1
Friend Amount	2.89	0.999	5	1	5	3	1
Group Contact	2.67	0.945	5	1	4	3	1
Psychological Capital	4.2124	0.581	5	1	5	4.25	3.25

**Table 5:** The analysis results of effect quantity by leveraging the NCA method.

Condition	Method	Accuracy (%)	Upper Limit Region	Scope	Effect Size (d) ^a^	*p* Value ^b^
Education Level	CR	100%	0.003	0.776	0.004	0.503
	CE	100%	0.006	0.776	0.008	0.503
Sport Skill Level	CR	100%	0.000	0.87	0.000	1.000
	CE	100%	0.000	0.87	0.000	1.000
Vocational Skill	CR	99.9%	0.005	0.91	0.006	0.010
	CE	100%	0.006	0.91	0.006	0.011
Relative Help	CR	100%	0.000	0.87	0.000	1.000
	CE	100%	0.000	0.87	0.000	1.000
Friend Amount	CR	100%	0.004	0.87	0.005	0.001
	CE	100%	0.009	0.87	0.010	0.000
Group Contact	CR	100%	0.000	0.92	0.000	0.319
	CE	100%	0.001	0.92	0.001	0.319
Psychological Capital	CR	96.1%	0.029	0.92	0.031	0.009
	CE	100%	0.013	0.92	0.014	0.000

Note: ^a^: Effect size represents the fuzzy-set membership value after calibration, where 0.0≤d≤0.1 represents low level, 0.1≤d<0.3 denotes average, and 0.3≤d<0.5 refers to high level. ^b^: The p value is obtained utilizing the permutation test with 10,000 redraws in the NCA method.

**Table 6:** The analysis results of the bottleneck level, where NN represents an unnecessary condition (%).

Employment Quality Index	Education Level	Sport Skill Level	Vocational Skill	Relative Help	Friend Amount	Group Contact	Psychological Capital
0	NN	NN	NN	NN	NN	NN	NN
10	NN	NN	NN	NN	NN	NN	NN
20	NN	NN	NN	NN	NN	NN	NN
30	NN	NN	NN	NN	NN	NN	NN
40	NN	NN	NN	NN	NN	NN	NN
50	NN	NN	NN	NN	NN	NN	NN
60	NN	NN	NN	NN	NN	NN	NN
70	NN	NN	NN	NN	NN	NN	NN
80	NN	NN	NN	NN	NN	NN	5.3
90	13.1	NN	43.2	NN	35.7	9.5	15.0
100	25.6	NN	51.4	NN	47.8	13.7	24.7

**Table 7:** The necessity test results for individual variables.

Condition	High-Quality Employment		Non-High-Quality Employment	
	Consistency	Coverage Rate	Consistency	Coverage Rate
Education Level	0.870354	0.656013	0.843793	0.589907
No Education Level	0.455926	0.758850	0.507976	0.784216
Sport Skill Level	0.733512	0.664131	0.708243	0.594786
No Sport Skill Level	0.552457	0.671215	0.600067	0.676230
Vocational Skill	0.716425	0.656322	0.618308	0.525391
No Vocational Skill	0.481934	0.576502	0.595545	0.660785
Relative Help	0.600088	0.713634	0.547839	0.604289
No Relative Help	0.667248	0.614042	0.740383	0.631974
Friend Amount	0.525598	0.733773	0.460238	0.595967
No Friend Amount	0.710591	0.586663	0.794402	0.608332
Group Contact	0.653778	0.732185	0.589687	0.612553
No Group Contact	0.654037	0.632147	0.742175	0.665356
Psychological Capital	0.664900	0.673651	0.620129	0.582764
No Psychological Capital	0.588180	0.625373	0.652723	0.643708

## Data Availability

Data are available in a publicly accessible repository https://github.com/zouhaochen/Research-on-the-realization-path-of-high-quality-employment-for-retired-athletes, accessed on 24 February 2026.
